# Mercury-Selenium Relationships in Liver of Guiana Dolphin: The Possible Role of Kupffer Cells in the Detoxification Process by Tiemannite Formation

**DOI:** 10.1371/journal.pone.0042162

**Published:** 2012-07-31

**Authors:** José Lailson-Brito, Renato Cruz, Paulo Renato Dorneles, Leonardo Andrade, Alexandre de Freitas Azevedo, Ana Bernadete Fragoso, Lara Gama Vidal, Marianna Badini Costa, Tatiana Lemos Bisi, Ronaldo Almeida, Dario Pires Carvalho, Wanderley Rodrigues Bastos, Olaf Malm

**Affiliations:** 1 Laboratório de Mamíferos Aquáticos e Bioindicadores “Profa. Izabel Gurgel” (MAQUA), Faculdade de Oceanografia, Universidade do Estado do Rio de Janeiro (UERJ), Rio de Janeiro, Rio de Janeiro, Brazil; 2 Laboratório de Radioisótopos “Eduardo Penna Franca”, Instituto de Biofísica Carlos Chagas Filho, Universidade Federal do Rio de Janeiro (UFRJ), Rio de Janeiro, Rio de Janeiro, Brazil; 3 Departamento de Biologia Celular e Molecular, Universidade Federal Fluminense (UFF), Niterói, Rio de Janeiro, Rio de Janeiro, Brazil; 4 Instituto de Ciências Biomédicas, Universidade Federal do Rio de Janeiro (UFRJ), Rio de Janeiro, Rio de Janeiro, Brazil; 5 Instituto de Ciências Exatas e Naturais, Universidade do Estado do Rio Grande do Norte (UERN), Mossoró, Rio Grande do Norte, Brazil; 6 Laboratório de Biogeoquímica Ambiental, Universidade Federal de Rondônia (UNIR), Porto Velho, Rondônia, Brazil; IIT Research Institute, United States of America

## Abstract

Top marine predators present high mercury concentrations in their tissues as consequence of biomagnification of the most toxic form of this metal, methylmercury (MeHg). The present study concerns mercury accumulation by Guiana dolphins (*Sotalia guianensis*), highlighting the selenium-mediated methylmercury detoxification process. Liver samples from 19 dolphins incidentally captured within Guanabara Bay (Rio de Janeiro State, Brazil) from 1994 to 2006 were analyzed for total mercury (THg), methylmercury (MeHg), total organic mercury (TOrgHg) and selenium (Se). X-ray microanalyses were also performed. The specimens, including from fetuses to 30-year-old dolphins, comprising 8 females and 11 males, presented high THg (0.53–132 µg/g wet wt.) and Se concentrations (0.17–74.8 µg/g wet wt.). Correlations between THg, MeHg, TOrgHg and Se were verified with age (p<0.05), as well as a high and positive correlation was observed between molar concentrations of Hg and Se (p<0.05). Negative correlations were observed between THg and the percentage of MeHg contribution to THg (p<0.05), which represents a consequence of the selenium-mediated methylmercury detoxification process. Accumulation of Se-Hg amorphous crystals in Kupffer Cells was demonstrated through ultra-structural analysis, which shows that Guiana dolphin is capable of carrying out the demethylation process via mercury selenide formation.

## Introduction

In general, apex predators present high mercury concentrations in their tissues due to the biomagnification of the most toxic form of the metal, methylmercury (MeHg) [1]. Mammals absorve mercury through skin contact and inhalation, as well as via placental or lactational transfer; however, the main exposure occurs through diet [2], [3], [4].

Studies have shown high mercury accumulation in aquatic mammal tissues [2], [3], [5], [6]. This micropollutant is well known for its neuro- and immunotoxic effects on wildlife, and its toxicity to marine mammals was established through in vitro studies [7], [8], [9], [10]. Additionally, some investigations have tried to link trace metal concentrations in free-ranging marine mammals and health status [11], [12], [13]. However, there are several studies that have not found adverse effects related to mercury toxic action in marine mammals [5]. Such observation seems to be associated with the protective action of metallothioneins, as well as with mercury-selenium interactions, through the formation of mercuric selenide [14], [15]. Stoichiometric relations between mercury and selenium have been reported for marine mammal liver since 1970s and these relations have been attributed to methylmercury detoxification processes [16], [17], [18].

The Guiana dolphin (*Sotalia guianensis*) can be useful as a model for the comprehension of mercury accumulation and methylmercury detoxification processes. The species inhabits coastal zones, which implies that Guiana dolphins are in constant interaction with anthropogenic activities. This is specially important in Guanabara Bay, the most degraded area along the species distribution [19], [20], [21], [22], [23]. This bay constitutes the marine environment under the highest anthropogenic pressure along the Brazilian coast [24]. An assessed population of 14 million people lives in the 12 cities of its drainage basin and 35 rivers flow into the bay. These cities house 6 000 industrial plants. In addition, 500 tons of “in natura” sewage and 6.9 tons of oil are released daily into the bay [25], [26]. Regarding heavy metal contamination of this body of water, the elements that raise the highest concern are Cr, Pb, Cu and Hg [27], [28], [29]. Other sources of metals, such as atmospheric deposition, also contribute significantly to the total metal burden that enters Guanabara Bay [27], [29]. Recent studies have shown Guiana dolphin presents residence pattern in bays and estuaries along its distribution [30], [31], which emphasizes its usefulness as sentinel of environmental pollution in coastal tropical ecosystems from West Atlantic.

The present study aimed to investigate the methylmercury (MeHg) detoxification in hepatic tissue of Guiana dolphins through the relationships among total mercury (THg), total organic mercury (TOrgHg), MeHg and selenium (Se). Additionally, intracellular sites of mercury accumulation were identified in hepatic tissue through ultrastructural histological investigations, reveling a possible route for MeHg detoxification.

## Materials and Methods

### Ethics Statement

Liver samples of Guiana dolphins were collected with appropriate permissions from Brazilian Environmental Agencies – IBAMA/MMA (permission number 11495-1) and ICMBio/MMA (permission number 11579-1).

### Sampling

Hepatic tissue samples were obtained from Guiana dolphins, *Sotalia guianensis*, that were incidentally captured in gillnet fishery off Rio de Janeiro coast, from 1994 to 2006. All samples were stored in individual polyethylene bags and kept frozen (−20°C) until analysis. Nineteen dolphins, including 11 males and 8 females, were analyzed. Age was determined by counting the growth layer groups present in dentine and cementum of the teeth [32] and varied from 0 (fetus and neonate) up to 30 years. Total length (TL) ranged from 71 to 198 cm (Table 1).

**Table 1:** Biological information and analyte concentrations in liver of Guiana dolphins.

Specimen	Sex	TL^1^	Age^2^	THg^3^	THg^4^	MeHg^5^	%MeHg^6^	TOrgHg^7^	Se^8^	Se^9^	Se:THg^10^
Sg #01	F	71.0	0	2.52	12.5	0.48	19.0	0.50	4.51	57.1	4.55
Sg #02	F	73.0	0	11.8	58.7	0.11	0.93	0.62	74.8	948	16.1
Sg #03	F	75.0	0	3.80	18.9	0.31	8.15	0.56	0.17	2.14	0.11
Sg #04	M	92.5	0	1.33	6.65	0.38	28.6	0.42	0.65	8.25	1.24
Sg #05	M	133.0	1	2.26	11.2	0.91	40.3	1.33	0.36	4.56	0.40
Sg #06	M	147.5	1	0.59	2.93	0.56	94.9	0.66	nd^11^	nd^11^	nd^11^
Sg #07	M	176.0	4	2.51	12.5	0.78	31.1	0.93	nd^11^	nd^11^	nd^11^
Sg #08	M	177.0	8	4.60	22.9	0.20	4.35	0.89	0.64	8.11	0.35
Sg #09	F	185.0	8	5.68	28.3	0.60	10.6	1.99	1.16	14.6	0.52
Sg #10	M	184.5	9	17.3	86.4	nd^11^	nd^11^	1.00	6.56	83.1	0.96
Sg #11	M	189.0	13	24.7	123	0.78	3.15	1.16	nd^11^	nd^11^	nd^11^
Sg #12	M	190.0	13	47.6	237	1.35	2.83	1.58	15.3	193	0.81
Sg #13	F	198.0	23	132	661	1.74	1.31	1.86	53.9	682	1.03
Sg #14	F	180.0	30	41.7	208	1.84	4.41	nd^11^	21.2	268	1.29
Sg #15	F	185.0	30	60.1	299.6	1.92	3.19	nd^11^	nd^11^	nd^11^	nd^11^
Sg #16	F	149.0	nd^11^	0.53	2.63	nd^11^	nd^11^	nd^11^	0.23	2.87	1.09
Sg #17	M	187.0	nd^11^	13.5	67.3	0.95	7.03	1.12	nd^11^	nd^11^	nd^11^
Sg #18	M	187.0	nd^11^	3.57	17.8	0.45	12.6	0.63	1.59	20.1	1.13
Sg #19	M	189.0	nd^11^	3.69	18.4	1.22	33.1	1.43	2.01	25.4	1.38

1 Total length in cm.

2 Age in years.

3 Total mercury concentration in µg/g, wet wt.

4 Total mercury concentration in nmol/g, wet wt.

5 Methylmercury concentration in µg/g, wet wt.

6 Percentage of methylmercury contribution to total mercury.

7 Total organic mercury concentration in µg/g, wet wt.

8 Selenium concentration in µg/g, wet wt.

9 Selenium concentration in nmol/g, wet wt.

10 Selenium: total mercury molar ratio.

11 Not determined.

### Se Determination

Aliquots of approximately 200 mg of liver were digested with 2 mL of 65% HNO_3_ in a screw-capped vessel, during 24 h. The solution was then heated to 60°C for 120 min in a water bath. Total selenium was determined by electrothermal atomic absorption spectrometry (ET-AAS), using an Analytik Jena spectrometer ZEEnit 60 equipped with Zeeman-effect background correction. Palladium nitrate was used as a matrix modifier and hence added to each solution to be analyzed. Standard solutions in the working range (20–100 ng/mL) were prepared from Se stock solution (1000 mg/L), using 13% v/v HNO_3_. Quality control was carried out using blanks, as well as, standard reference material DOLT-2 (NRC, Canada). Our analytical results (in µg/g wet wt. ±S.D.) for the determination of selenium in DOLT-2 (5.94±0.07 µg/g) were in good agreement with certified value (6.06±0.49 µg/g). The detection limit of the method was 0.00326 µg/g.

### THg Determination

Aliquots of approximately 200 mg of liver were weighted in a vessel in which 1 mL of H_2_O_2_ was added. After this first step, the addition of 5 mL of an acid mixture of HNO_3_ and H_2_SO_4_ (1∶1) was performed and the solution was then heated to 60°C for 120 minutes in a water bath. After cooling during 15 minutes, the addition of 5 mL of KMnO_4_ (5%) was executed. The samples went through a new step of heating in the water bath (60°C) for 15 minutes before overnight digestion stage. The day after, hydroxylamine hydrochloride (1 mL) was added to the samples. Total mercury determination was performed using an Atomic Absorption Spectrophotometer for Hg measurement with Flow Injection System (FIMS-400, Perkin Elmer). Blanks and standard reference material (DOLT-2 – NRC, Canada) were carried through the procedure in the same way as the sample. Our analytical results (in µg/g wet wt. ±S.D.) for mercury determination in DOLT-2 (2.16±0.08 µg/g) were in good agreement with certified value (2.14±0.28 µg/g). The detection limit of the method was 0.0017 µg/g.

### MeHg Determination

Aliquots of approximately 50 mg of liver were put in a stove 68°C during 3–4 hours for samples lixiviation [33], [34], [35]. The sample was digested in an alcaline solution with 25% KOH/methanol degree HPLC and then made up to a known volume with high purity deionised water (18.2 MΩ cm) from a Milli-Q system. After extraction, 30 µL from the sample were transferred to an amber vessel and 200 µL of buffer solution (NaC_2_H_3_O_2_ 2 M) were added to adjust to pH 4.9. The solution was then made up to 40 mL with Milli-Q water. After that, the samples were ethylated by the addition of 50 µl of NaBEt_4._ MeHg was quantified on a GC-AFS (MERX™ Automated Methyl Mercury Analytical System, Brooks Rand) [36]. Our analytical results for MeHg determination in DOLT-2 (0.671 µg/g) were in good agreement with certified value (0.693 µg/g). The detection limit of the method was 0.0005 µg/g.

### TOrgHg Determination

TOrgHg determination was conducted according to Uthe et al. [37] and Wagemann et al. [38]. Samples were mixed with an aqueous solution of acid potassium bromide (30% in 4 N H_2_SO_4_) and cupric sulfate (2.5% in 4 N H_2_SO_4_) to release all the organic forms of mercury, and then extracted into dichloromethane (DMC) and N-hexane phase. An aliquot (1 mL) of the organic phase was removed and then heated with a mixture of acids (HNO_3_-H_2_SO_4_; 1∶4 v/v) in water bath for 30 min at 60°C. The remaining phase was digested and analyzed for total mercury as explained above. Our analytical results for MeHg determination (used as reference to TOrgHg) in DORM-3 (NRCC) were (0.388 µg/g) in good agreement with certified value (0.355 µg/g).

### Optical Microscopy

During dolphin necropsies, different hepatic tissue fragments (5 mm) were transferred to vials containing 10% paraformaldehyde (EMS) in 0.1 M sodium phosphate buffer (pH 7.2) for 2 hours. The tissue was then rinsed in the same buffer twice for 15 minutes, dehydrated in graded ethanol series (50, 70, 90 and 100%) for 15 minutes each bath. After dehydrating, the ethanol was replaced with xylene solutions and the tissue was embedded in paraffin. Semi-thin sections (6 µm) were obtained with histological razor blade (Ted Pella) in a Leica microtome and the sections were stained with hematoxilin & eosine (HE). The sections were observed in a Jenaval Universal (Carl Zeiss-Jena) equipped with a digital camera (Sony, Cybershot DSC-P93). The images (1024×960 pixels) were acquired in bright field and under polaryzed light with crossed Polaroids.

### Conventional and Analytical Transmission Electron Microcopy

For ultrastructural observations and X-ray microanalysis, samples were fixed in 2.5% glutaraldehyde (Sigma, EM grade), in 0.1 M sodium cacodylate buffer (pH 7.4), as well as in filtered seawater for 2 hours. Then, the samples were rinsed in the same buffer twice for 15 minutes. Samples were not post-fixed in osmium tetroxide to avoid undesirable X-ray peaks. The tissue fragments were dehydrated in graded acetone series (50, 70, 90, 100%) for 15 minutes each bath and embedded in Spurr resin (Ted Pella). Ultra-thin sections (90 nm) were obtained in an ultramicrotome (Sorval) and transferred to copper grids (300 mesh). The ultra-thin sections were observed in a Zeiss EM 906 and operated at 80 kV. X-ray microanalysis was performed in a Jeol 1200 EX equipped with a Noran-Voyager analytical system.

### Statistical Treatment

Since Shapiro-Wilk’s W test indicated that the data did not present normal distribution, non-parametric tests were used. The Spearman’s (Rs) correlation test was used for investigating the occurrence of correlation between the concentrations of each element or compound and the variables total length and age; as well as for testing correlations between Se and THg, and between the percentage of MeHg in THg and THg concentration. All statistical analyses were performed using the software Statistica (version 7, StatSoft, Inc.).

## Results and Discussion

THg, TOrgHg, MeHg and Se concentrations (µg/g wet wt.) in livers of Guiana dolphins are presented in the Table 1.

### Mercury Accumulation

Hepatic THg concentrations of Guiana dolphins varied from 0.53 to 132 µg/g wet wt. A similar range for THg (0.35 to 95.0 µg/g wet wt.) in liver samples of Guiana dolphins from southern São Paulo and northern Paraná State was reported [39]. However, Guiana dolphins from Ceará State (northeast Brazil) presented apparently lower hepatic Hg concentrations (0.10 to 29.5 µg/g wet wt.) [40]. Comparing to other small coastal odontocetes, hepatic THg concentrations found in the present study were higher than those observed in harbor porpoises (*Phocoena phocoena*) from North Atlantic (3.00 to 26.0 µg/g dry wt.) [41], but similar to those hepatic concentrations verified in Indo-Pacific humpback dolphins (*Souza chinensis*) from waters of Hong Kong (from <0.15 to 228 µg/g wet wt.) [42].

**Figure 1:**
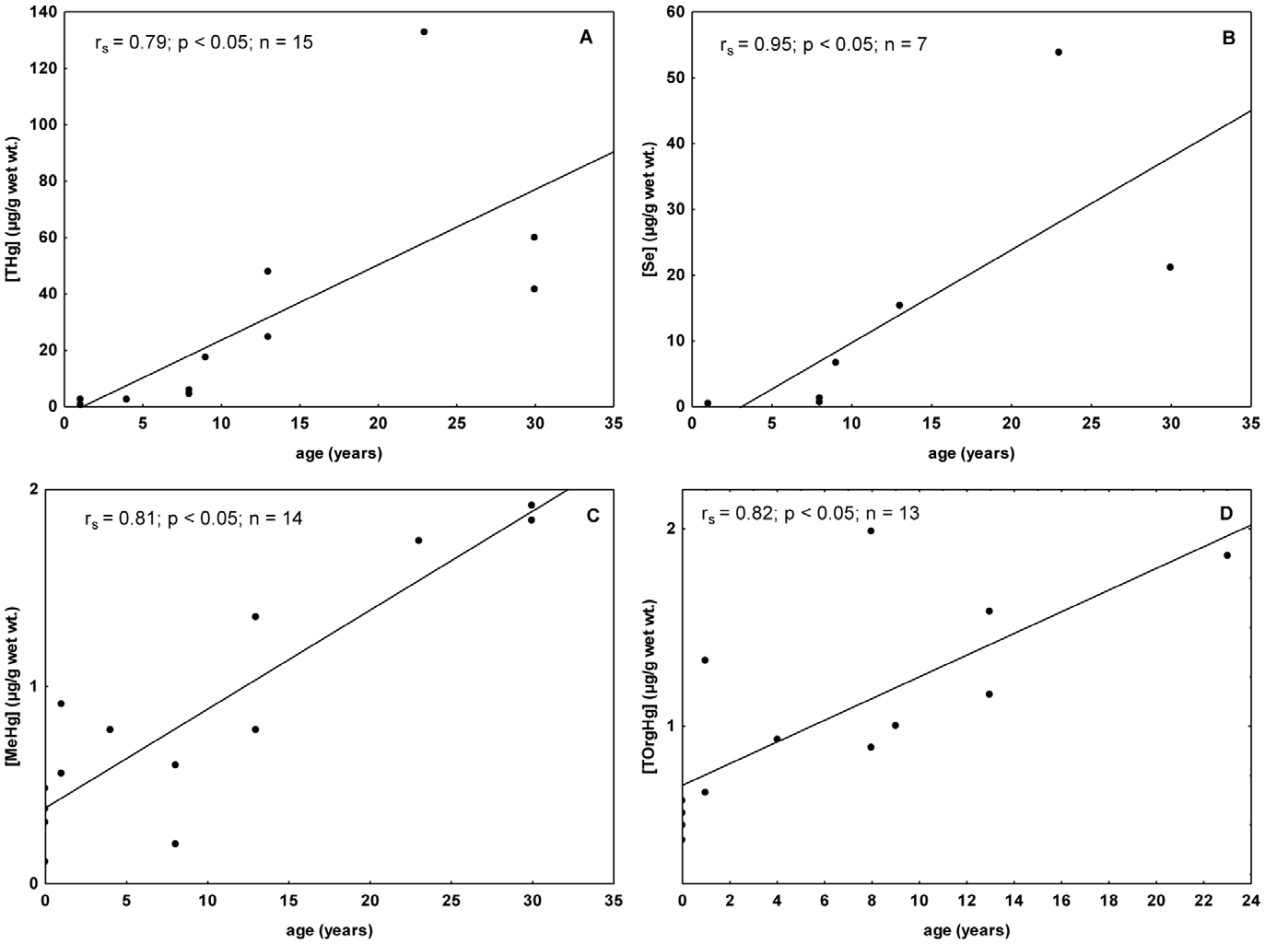
Spearman correlations between hepatic concentrations of THg, Se, MeHg, TOrgHg and age of Guiana dolphins. The specimens are from Guanabara Bay, Rio de Janeiro State. Concentrations are in µg/g wet wt. and age in years. (A) Relationships between total mercury concentration (THg) and age. (B) Relationships between selenium concentration (Se) and age - data from fetus and neonates were excluded from the analysis. (C) Relationships between methylmercury concentration (MeHg) and age. (D) Relationships between total organic mercury (TOrgHg) and age.

THg was significantly positively correlated with total length (Spearman correlations; r_s_ = 0.61, p<0.05), which seems to be a result of Hg accumulation with the age of Guiana dolphins (Spearman correlations; r_s_ = 0.79, p<0.05) (Fig. 1A). An increasing THg concentration with age constitutes a pattern reported for marine mammals from all over the world, including odontocete and mysticete cetaceans [4], [39], [43]. This probably occur due to the high biological half-life of metals that present high affinity for the cysteine SH group (Cd, Hg, Pb and Ag) [44].

Maternal transfer of mercury is known to occur in marine mammals [4] and fetus/mother ratio of hepatic THg concentration may be used for comprehension on the placental transfer of this metal [45]. Fetus/mother ratios for two Guiana dolphin pairs were 0.19 and 0.03, demonstrating occurrence of restrict transplacental transfer of Hg in the species. These ratios were comparable with Honda et al. [45], who found ratios lower than 1.0. Hg transfer during pregnancy was estimated varying from 0.4 to 1.0% for striped dolphin, *Stenella coeruleoalba* [46]. The low values observed for this ratio both in the present study and in literature suggest the existence of a certain restriction in this transfer. Born et al. [47] reported similar concentrations in newborn walrus and their mothers (*Odobenus rosmarus rosmarus*), which would indicate a higher Hg transfer efficiency through milk than via placenta. However, it was recently suggested that, in northern elephant seals (*Mirounga angustirostris*), the maternal transfer of mercury was larger during gestation than during lactation [48]. Similar result was previously reported for harp seal pups (*Phoca groenlandica*), which received much of the mercury as methylmercury [49].

One lactating (Sg#14) and two pregnant (Sg#13 and Sg#15) females presented the highest THg concentrations among the analyzed Guiana dolphins. Caurant et al. [14] also observed the highest THg concentrations in lactating females and the authors attributed this finding to a feeding habit alteration due to a higher energetic demand. However, it has been observed that pregnant females or iron-deficient organisms tend to undergo an increase in their non-essential elements gastrointestinal absorption rate, such as Cd [50]. Cd and Hg are from the group IIB metals, which could indicate the existence of a similar behavior for these elements. Hence, there could be an enrichment of inorganic Hg during pregnancy.

### Selenium-mercury Relationships and Their Role in Methylmercury Detoxification Processes

Significant positive correlations were found between hepatic selenium concentrations and both age and total length when fetus and neonates were excluded from the analysis (Spearman correlations, p<0.05) (Fig. 1B). This pattern has been verified in a number of aquatic mammal species [14], [15], [51]. Fish and cephalopods may constitute important source of selenium for predator organisms, as it is the case of two teleost fish that comprise the diet of Guiana dolphins, mullet (*Mugil* sp.) and whitemouth croaker (*Micropogonias furnieri*) [52].

Aquatic mammals accumulate high Hg concentrations in their tissues, particularly in liver. Despite this common finding, investigations that describe deleterious effects of mercury on the health of marine mammals are scarce [11], [53]. However, it was reported that mercury, selenium and zinc concentrations, as well as Hg:Se ratios, were significantly higher in harbor porpoises that died from infectious diseases than in those individuals that died as consequence of physical trauma [12]. In general, it has been argued that the molar ratio between the elements is more important than the concentration itself. It is clear that understanding the mercury-selenium relationship is of fundamental importance for the comprehension of the methylmercury detoxification processes, as well as for the understanding of the processes related to mercury immobilization under a non-toxic form [18], [54], [55]. Several studies have reported Hg-Se molar ratios close to 1.0 in marine mammal liver [17], [56], [57], [58], including an investigation that dealt with samples from two Guiana dolphins from Suriname [56]. Koeman et al. [17], [56] suggested a causal relationship between both elements, which would be involved in a detoxification process. Additionally, Lemes et al. [55] have detected methylmercuric glutathionate (CH_3_HgSG) and Hg-Se complexes in liver and brain tissues of beluga whales from the western Canadian Arctic. The authors suggest that these compounds are associated to the protection against the toxic effect of the high concentrations of MeHg found in beluga whales [55].

**Figure 2:**
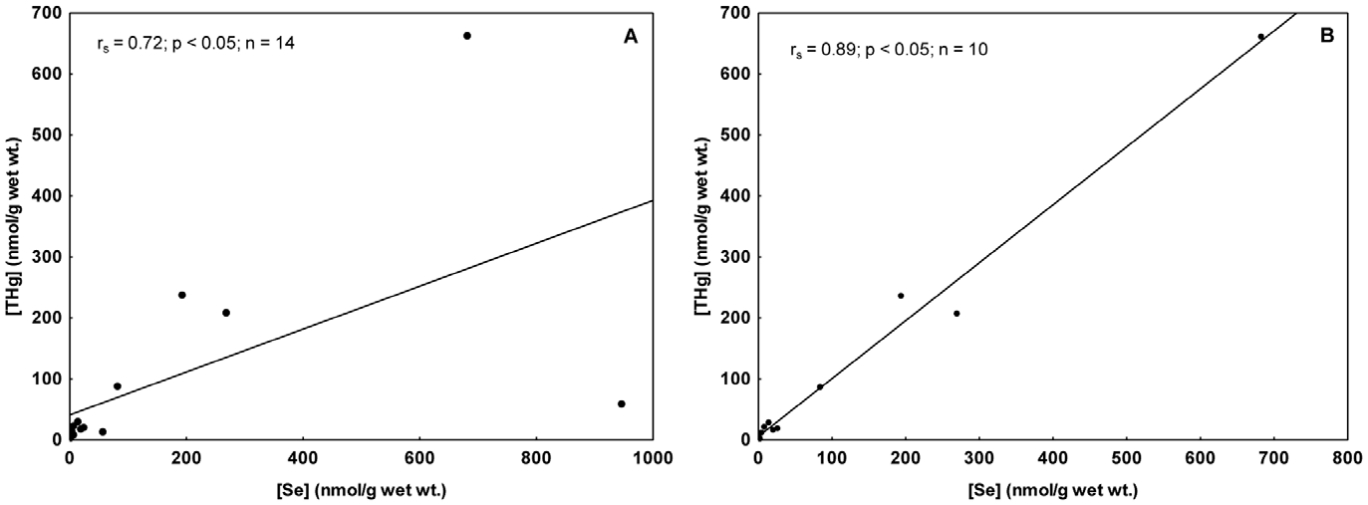
Spearman correlations between THg and Se molar concentrations in liver of Guiana dolphins. The specimens are from Guanabara Bay, Rio de Janeiro State. THg and Se concentrations are in nmol/g wet wt. (A) Relationship between total mercury and selenium considering all individuals. (B) Relationship between total mercury and selenium excluding data from fetus and neonates.

In the present study, hepatic THg accumulation was followed by hepatic Se accumulation. The positive correlation coefficient of r_s_ = 0.72 (Spearman correlation, p<0.05; Fig. 2A) turned it possible to verify the latter finding. An increase in the correlation coefficient was observed when the data from fetuses and neonates were removed from the test (r_s_ = 0.89; Fig. 2B). During both of these early life stages, mammals may not present well-developed MeHg detoxification mechanisms, which alters the selenium-mercury relationship in comparison to adults. Another possible explanation is that Hg and Se may have different transfer efficiency. As discussed above, Habran et al. [48] verified that maternal transfer of Se was extended during lactation, whereas Hg transfer was more important during gestation in the northern elephant seal. The mean Hg-Se molar ratio was 2.2 and fetuses were the individuals that presented both the highest and the lowest Hg-Se molar ratios (0.11 e 16.1) among all dolphins analyzed (Table 1). A high transfer of both Hg and Se from mother (Sg#15) to fetus (Sg#2) constitutes a possible explanation for this wide variation. The specimen Sg#2 showed the highest Hg concentration among fetuses and calves and the highest Se concentration among all individuals.

Palmisano et al. [15] suggest Hg-Se molar ratio to get near 1.0 only in hepatic concentrations close to 100 µg/g in striped dolphins (*Stenella coeruleoalba*). However, it is important to highlight the mercury fraction used by the authors, since they neither used the protein-bound fraction nor they used MeHg (THg – (Hg^++^ bound to proteins + MeHg)). Considering that just few individuals presented hepatic Hg concentrations higher than 10.0 µg/g, our results may not be representative. However, our results indicated a trend for Hg-Se molar ratios close to 1.0 (Table 1). Palmisano et al. [15] did not find Hg concentrations between 10.0 and 100 µg/g. Therefore, it is possible that the Hg-Se molar ratio get near 1.0 even below the concentration suggested by the latter authors. This hypothesis agrees with the approximate limit of 50.0 µg/g suggested for another delphinid species, the long-finned pilot whale (*Globicephala melas*) [14].

**Figure 3:**
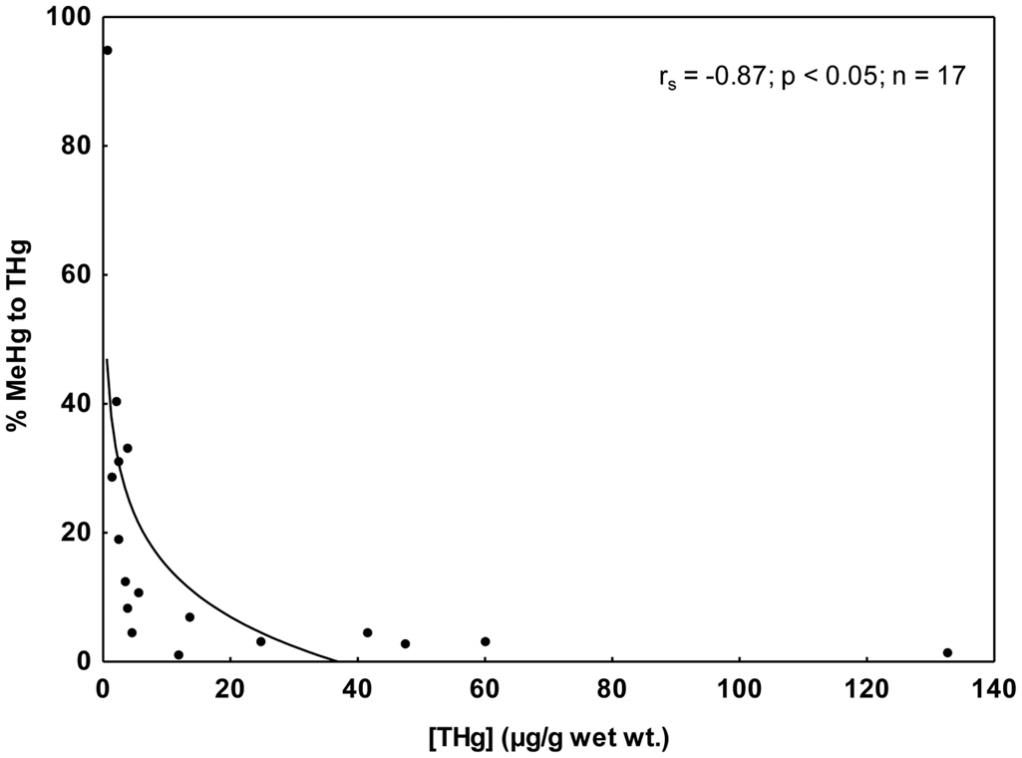
Spearman correlation between percentages of methylmercury (MeHg) to total mercury (THg) concentrations in liver samples from Guiana dolphin. The specimens are from Guanabara Bay, Rio de Janeiro State. THg concentration is in µg/g wet wt.

The occurrence of demethylation process seems to be responsible for the hepatic MeHg concentration. Low percentages of MeHg and TOrgHg contribution to THg were found in the present study (Table 1). This behavior had been described for Guiana dolphins from southern São Paulo and northern Paraná states regarding organic mercury [39], as well as, for other cetacean species [14], [15], [46], [49]. Additionally, the percentage of MeHg to THg tend to decrease with the increase of THg in livers of Guiana dolphins (Spearman correlations; r_s_ = −0.87, p<0.05) (Fig. 3). Apparently, as MeHg suffers demethylation, Hg is immobilized under an inorganic form. Therefore, the percentage of MeHg contribution to THg tends to reduce due to the high rates of inorganic Hg accumulation.

Young cetaceans and pinnipeds do not seem to be able to demethylate MeHg efficiently [14], [46], [49], [59]. In general, marine mammal calves and fetuses present higher percentages of MeHg contribution to THg than their mothers. In the present study, the mother and the fetus (Sg#13 and Sg#3) presented the percentages of 1.3 and 8.2, respectively, which corroborates the general observation. Fetuses and one-year-old calves presented percentages of MeHg contribution to THg varying from 0.93 to 94.9%. Moreover, higher mean values were observed in fetuses and calves than in adults (32×13%), which constitute a pattern verified in other marine mammal species [14], [46], [49]. *In vitro* studies performed with hepatocytes from gray seal pups (*Halichoerus grypus*) did not evidence biochemical MeHg demethylation, whereas gray seal adults seem to demethylate MeHg [60]. It is interesting to note that the fetus (Sg#2) that showed the lowest percentage of MeHg contribution to THg was the specimen that presented the highest Hg-Se molar ratio (16.1). The mother (Sg#15) showed the highest THg concentration (299 µg/g wet wt.) and the percentage of MeHg contribution to THg was relatively low (3.19%). As previously discussed, the percentage of MeHg contribution to THg tends to reduce due to the high rates of inorganic Hg accumulation as result of the MeHg demethylation. Consequently, a high percentage of the THg transferred from the mother to the fetus was probably inorganic mercury.

In general, positive correlations were found between TOrgHg and age [2], [39] as observed to Guiana dolphins for both MeHg and TOrgHg concentrations (Spearman correlations; p<0.05; Figure 1C and 1D). Dietz et al. [59] suggested that MeHg concentrations seldom exceed 2.00 µg/g in health animals, which was corroborated by results obtained in other studies [3], [61]. Since MeHg presents a high positive correlation with age, Wagemann et al. [3] suggested that the demethylation rate reduces during the aging process. Our results lead to the hypothesis that the demethylation continues in a high rate in Guiana dolphins, as well as to the supposition that the process does not allow occurrence of higher concentrations than the mentioned threshold (2.0 µg/g). A plausible explanation for that relies on the fact that the liver of marine mammals is normally capable of keeping MeHg in low levels [59].

Regarding the percentage of MeHg contribution to TOrgHg, the values varied from 17.8 to 96.0% (mean value = 70%). This finding showed the TOrgHg concentration included organomercurial species other than MeHg [38], [62]. An arithmetic mean of 23% of MeHg contribution to TOrgHg was found in liver of ringed seals (*Phoca hispida*) [38], which was about three times lower than the MeHg percentage found in the present study.

### Kupffer Cells in the Detoxification Process by Tiemannite Formation

The presence of Hg under an insoluble form (mercury selenide granules - tiemannite) in marine mammal liver was suggested first by Koeman et al. [17]. Later, the accumulation of mercury selenide granules as amorphous crystals was demonstrated in hepatic tissue of Cuvier’s beaked whales [63], [64]. André et al. [65] suggested these particles do not suffer proteolytic enzyme attack and stay inert. The tiemannite accumulation was showed in liver, muscle and brain [57], as well as these crystals were identified in lung [66], [67].

**Figure 4:**
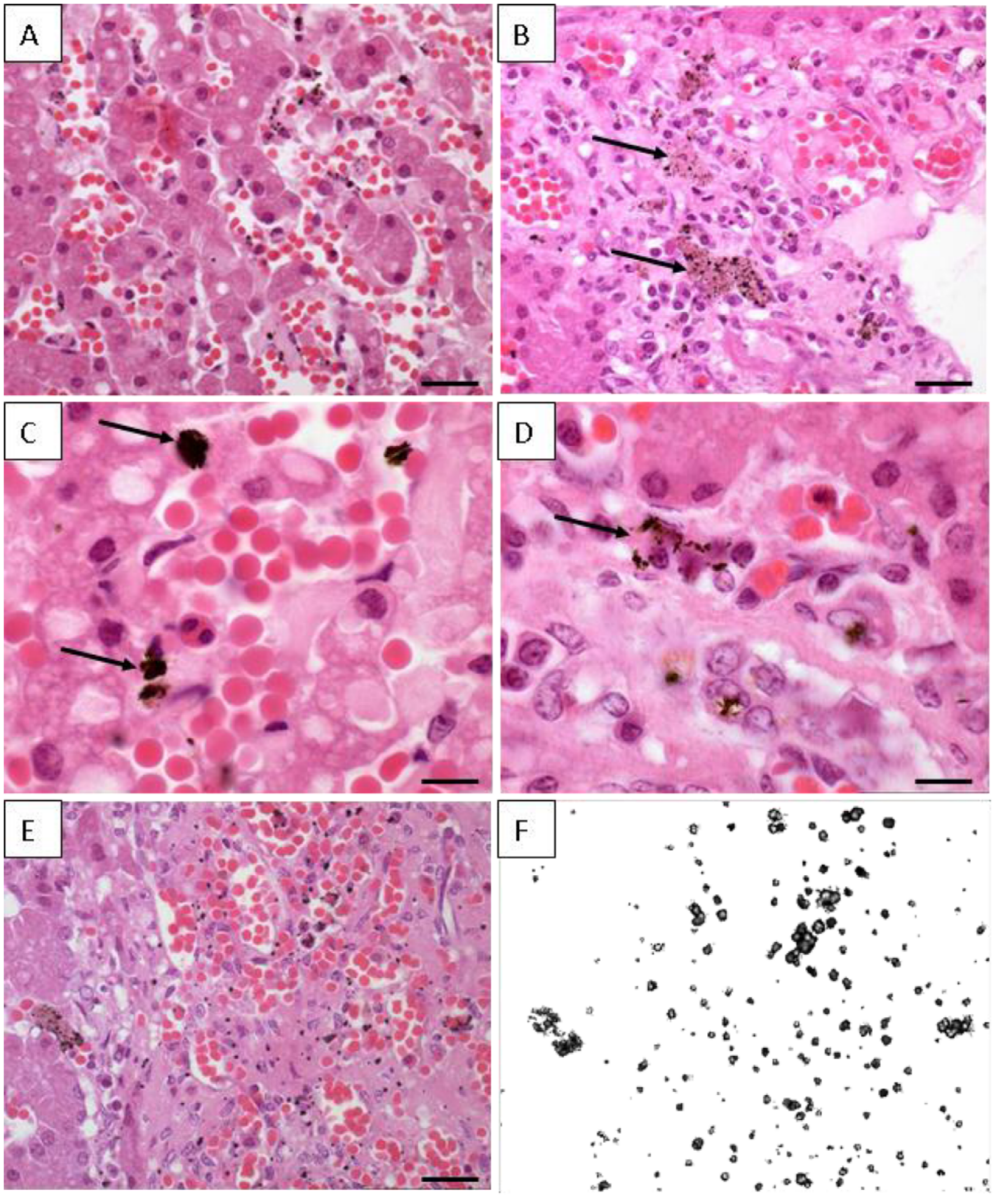
Histological sections from hepatic tissue of Guiana dolphin (*Sotalia guianensis*). The sample is from a 9-year-old male dolphin from Guanabara Bay, Rio de Janeiro State. (A) Semi-thin section of the hepatic tissue showing the hepatocytes arranged in layers or plates; Longitudinal blood vessel filled with numerous red cells can be seen; Barr = 80 µm. (B) Different field of the organ showing the hepatic parenchyma with many blood vessel and some dark deposits within the Kupffer cells (arrows); Barr = 100 µm. (C) Agglomerated dark deposits found in Kupffer cells (arrows); Barr = 20 µm. (D) Deposits distributed in small dots (arrows); Barr = 20 µm. (E) Semi-thin section of dolphin liver showing the cells with dark inclusions; Barr = 100 µm. (F) - Same area depicted in figure E observed under polaryzed light; all the dark dots are birefringents materials and corresponds to deposits within the Kupffer cells.

In the present study, histological and ultrastructural investigations have been performed. The hepatic tissue originated from a 9-year-old dolphin (17.3 µgTHg/g) presented the basic appearance of a normal liver, with hepatic parenchyma formed by hepatic lobules in a prism form, surrounded by connective tissue. The lobules were formed by plates of hepatocytes separated by hepatic sinusoids that radiate out from a central vein. Hepatocytes are polyhedral cells that fill the most volume of hepatic parenchyma. Sinusoids walls are lined by flattened endothelial cells interspersed with plumper Kupffer cells that often presented dark deposits (Fig. 4A and 4B). These deposits had different sizes and they can be seen as agglomerated or small dots (Fig. 4C and 4D). These materials within Kupffer cells, when observed under polarized light, were birefringents, indicating a crystalline nature (Fig. 4E and 4F).

The hepatic sinusoids are located adjacent to the hepatocytes, allowing the exchange of soluble substances between blood and hepatocytes. The Kupffer cells are derived from bone marrow monocytes and contain numerous endocytic vesicles, lysosomes and usually fagocytosed particulate matter. The dark vesicles found in our sample resembled lysosomes typical from those cells. In addition, these cells act as macrophages, removing virus, bacteria, tumor cells and parasites from the blood stream. A possible explanation is that Hg and Se diffused through the sinusoids, were absorbed and formed a crystalline mineral that remained insoluble within the Kupffer cells. Therefore, it is plausible to assume that besides being produced from the hepatic demethylation process, the Se-Hg crystal produced in other tissues is also accumulated in liver by the action of Kupffer cells, since it finds its way to the blood stream.

**Figure 5:**
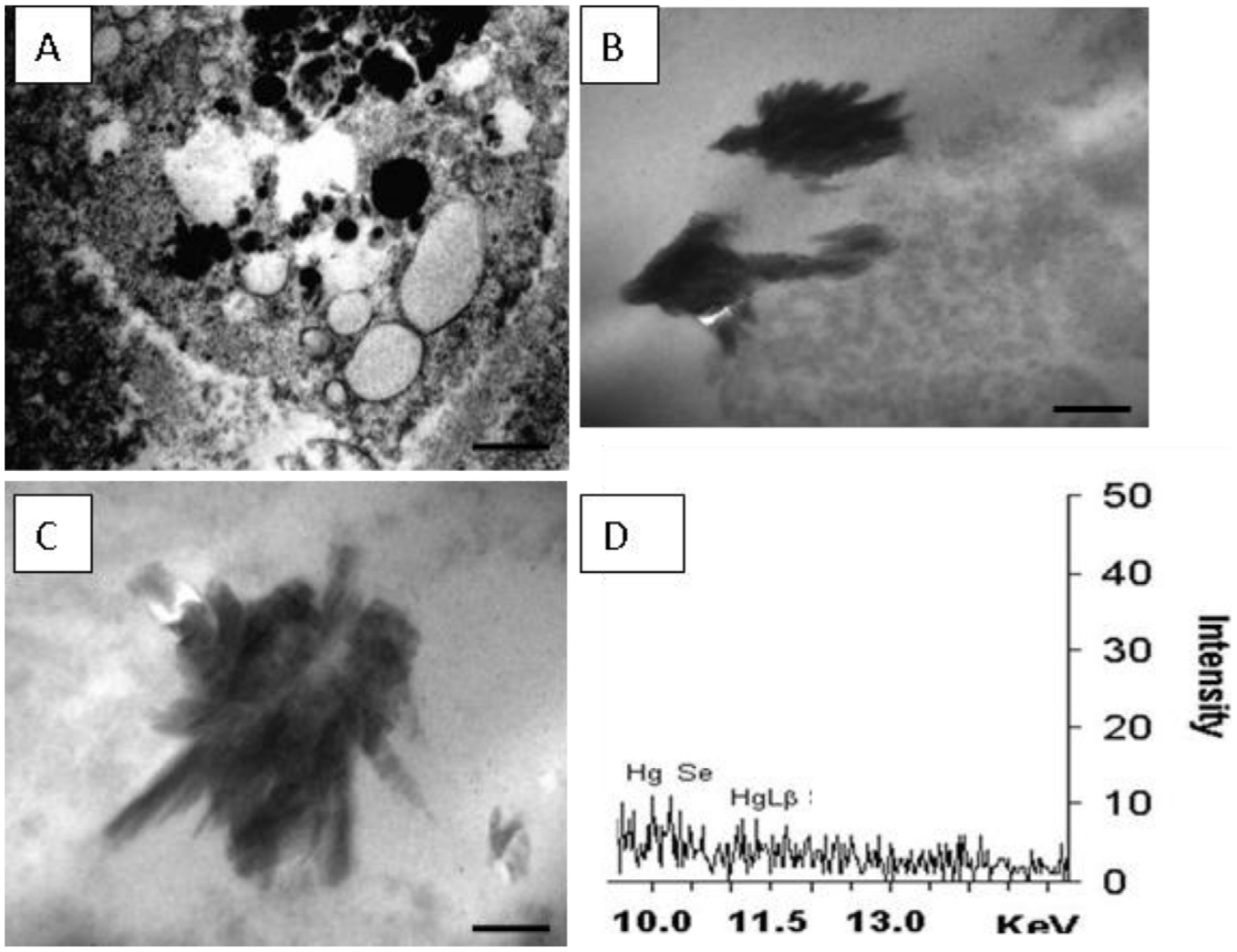
Ultrastructure of Guiana dolphin (*Sotalia guianensis*) liver. The sample is from a 9-year-old male dolphin from Guanabara Bay, Rio de Janeiro State. (A) Electron microcopy image showing a Kupffer cell and its organelles. Numerous circular vesicles, some electron-denses, other electron-lucents; Barr = 5 µm. (B) Image of stick-like shaped electron-dense deposits found in Kupffer cells; Barr = 1 µm. (C) A detail of other electron-dense deposit; Barr = 500 nm. (D) X-ray microanalysis spectra showing the elements Hg and Se as components of those dark deposits found within Kupffer cells.

Transmission electron microscopy of Kupffer cells showed numerous circular vesicles, some electron-denses, other electron-lucents (Fig. 5A). In addition, stick-like shaped electron-dense deposits (approximately 2 µm) were also seen (Fig. 5B and 5C). These deposits were not found within the hepatocytes. They were analyzed by X-ray microanalysis and Hg (Lα and Lβ layers) and Se (Kα layer) were detected co-localizing in the same studied region (Fig. 5D).

It is important to highlight that the low signal might have a straight relationship with the option for the tissue site to be analyzed. Priority was given to crystal analysis specifically rather than to crystal agglomerate, which certainly would produce an increase in the sign. However, the option for agglomerate would generate information not only on the crystal specifically but also on other structures that would mislead the interpretation of the results. Despite being close to the background noise, Hg and Se were the elements that could be highlighted, which did not happen by chance. Other abundant elements, such as copper, did not appear which clearly shows that the signal corresponds to the crystal rather than to the entire site. This finding suggests that Se-Hg granules are formed even in low concentrations.

Martoja and Berry [63] raised the hypothesis that tiemannite is a product of the biosynthesis process and that it represents the final stage of the MeHg degradation, suggesting that Se played a fundamental role in this process. The authors suggested also that tiemannite crystallization occurs in liver, since it is an organ in which the B12 vitamin is present in levels that allow the methyl group transfer from Hg to Se. This transfer would occur through dimethylselenide (CH_3_SeCH_3_) formation, which would be excreted by lungs. Nigro and Leonzio [57] corroborate such proposition suggesting that MeHg demethylation might occur in absence of biochemical process due to the high affinity between the two elements. The immobilization of Hg under the tiemannite form in cetacean liver, as well as in other top aquatic predators, represents the final stage of methylmercury biomagnification process. These insoluble deposits probably remain in liver for long periods in an integrative process, as proposed by Martoja and Berry [63].

In conclusion, through ultra-structural analyses, our study suggests the accumulation of Se-Hg amorphous crystals in Kupffer Cells. Our investigation shows a picture of a crystal that is probably tiemannite, exposing it in a separated view rather than showing the entire accumulation site. Finally, our findings show that Guiana dolphin is capable of carrying out the demethylation process via mercury selenide formation.
